# Combination of co-crystal and nanocrystal techniques to improve the solubility and dissolution rate of poorly soluble drugs

**DOI:** 10.1007/s11095-022-03243-9

**Published:** 2022-05-12

**Authors:** Zun Huang, Sven Staufenbiel, Roland Bodmeier

**Affiliations:** College of Pharmacy, Freie Universität Berlin, Kelchstr. 31, 12169 Berlin, Germany

**Keywords:** co-crystals, nanocrystals, nano-co-crystals, *in situ* methods, kinetic solubility

## Abstract

**Purpose:**

Solubility and dissolution rate are essential for the oral absorption and bioavailability of poorly soluble drugs. The aim of this study was to prepare nano-co-crystals by combination of nanocrystal and co-crystal technologies, and investigate its effect, *in situ*, on increased kinetic solubility and dissolution rate.

**Methods:**

Co-crystals of itraconazole-fumaric acid, itraconazole-succinic acid, indomethacin-saccharin and indomethacin-nicotinamide were prepared and nano-sized by wet milling. The particle size and solid state of the co-crystals were characterized by optical microscope, LD, PCS, DSC and XRPD before and after milling.

**Results:**

300-450 nm sized nano-co-crystals with a stable physical solid state were successfully prepared. Nano-co-crystals exhibited a lower crystallinity reduction than nanocrystals after wet milling. The particle size effect on the kinetic solubility of co-crystals was analysed for macro-, micro- and nano-co-crystals with *in situ* kinetic solubility studies. The maximum kinetic solubility of nano-co-crystals increased with excess conditions until a plateau. The highest increase was obtained with itraconazole-succinic acid nano-co-crystals with a kinetic solubility of 263.5 ± 3.9 μg/mL which was 51.5 and 6.6 times higher than the solubility of raw itraconazole and itraconazole-succinic acid co-crystal.

**Conclusions:**

The synergistic effect of nanocrystals and co-crystals with regard to increased kinetic solubility and dissolution rate was proven. The combination of the advantages of nanocrystals and co-crystals is a promising formulation strategy to increase both the solubility and dissolution rate of poorly soluble drugs.

**Supplementary Information:**

The online version contains supplementary material available at 10.1007/s11095-022-03243-9.

## Introduction

Active pharmaceutical ingredients (APIs) are categorized based on the Biopharmaceutics Classification System (BCS) indicating solubility and intestinal permeability as important factors for the rate and extent of oral bioavailability. Most new APIs are classified as poorly soluble in BCS class II. According to FDA guidance, an API is considered to be poorly soluble when the highest dose strength cannot be dissolved over the pH range of 1.0-6.8 in 250 mL aqueous media. The Developability Classification System (DCS) further divided BCS class II into IIa (dissolution-limited) and IIb (solubility-limited) based on the dose/solubility ratio, providing more useful information on suitable formulation strategies. Formulation approaches for improving IIa drugs focus primarily on improving dissolution rates by particle size reduction (1–3), whereas IIb drugs require an increased solubility, for example by formation of complexes (4), polymorphs (5), amorphous forms (6) and co-crystals (7).

Nanosuspensions (nanocrystals) consist of nano-sized pure drug particles and stabilizers. One of the most common approaches to nanocrystal preparation is wet bead milling. Nanocrystals improved oral bioavailability by increasing the dissolution rate of poorly soluble drugs (3, 8, 9). The dissolution rate is proportional to its surface area, thus reducing the particle size to nano range significantly increases the dissolution rate (10). However, the solubility enhancement by nanosuspensions of itraconazole, loviride, phenytoin and naproxen were reported with less than 15% (11). Another study reported that the saturation solubility of 300 nm dexamethasone and tacrolimus nanocrystals increased only by factor 1.3-2.8 compared with raw drug powders (12). The improvement in solubility was closely related to decreased crystallinity of the drug crystals after milling. However, the benefit of increased solubility on the basis of this decreased crystallinity was lost during storage, especially for those drugs with low glass transition temperature (13). Therefore, nanocrystal preparation might not be an effective method to increase drug solubility, where solubility was the rate-limiting step after oral administration.

A pharmaceutical co-crystal is composed of an API and an acceptable pharmaceutical molecule. Various methods of co-crystal preparation have been reported, e.g., solvent evaporation or reaction crystallization, grinding and fusion methods. Carbamazepine and saccharin co-crystals were prepared by neat grinding (14) and ibuprofen-nicotinamide co-crystals by hot melt extrusion (15). Itraconazole formed co-crystals with a number of C4 dicarboxylic acids by solvent evaporation method (16). The formation of co-crystals alters the original molecular arrangement and lattice packing of drugs, reduces lattice energy and improves drug solubility (17, 18). However, drug concentration in the co-crystal diffusion layer was significantly increased and the supersaturation occurs on the surface of the co-crystal during dissolution. Nucleation and crystal growth coincide with co-crystal dissolution and dissolved co-crystals transform into original crystal structure or precipitate on the surface of the co-crystal particles, preventing a rapid dissolution process and losing the advantage of co-crystal solubility (19, 20).

Combining co-crystals and nanocrystals could be a promising strategy to increase the solubility and dissolution rate of poorly soluble drugs by overcoming the limitation of each technique. Itraconazole, in combination with Tween 80 as stabilizer and excess adipic acid as coformer, was wet milled together at 150 rpm for 60 h to form nano-co-crystals with a particle size of 549 nm (21). Compared to a marketed drug product, this formulation yielded the same or faster release. Carbamazepine-saccharin, indomethacin-saccharin, and furosemide-caffeine nano-co-crystal suspensions were successfully prepared by wet milling of co-crystals with stabilizers; optimized nano-co-crystal suspensions were physically stable for at least one month and dissolution profiles were significantly improved (22). Phenazopyridine and phthalimide nano-co-crystals were prepared by a sonochemical anti-solvent precipitation method; release and oral bioavailability studies exhibited superior release rate and oral absorption compared to the hydrochloride salt and original co-crystal (23). However, robust preparation processes of nano-co-crystal formulations are still in the exploratory stage. Comparing wet bead milling and crystallization techniques to prepare nano-co-crystals, bead milling is the more robust method to prepare drug nanosuspensions including products that are being marketed. Milling media should be utilized in which milled compounds are poorly soluble and proper cooling during milling should be provided, reducing instability during and after preparation. However, reducing the crystallinity upon nano milling is possible whereas it should be investigated if milling of drug co-crystals instead of milling of raw drug powder could reduce this effect. In contrast, there is a high risk that nano-precipitation of dissolved co-crystals will result in an altered solid state besides a particle size which is difficult to control. Furthermore, the mechanism of increasing solubility and dissolution rate through the integration of nanocrystal and co-crystal technology has not been fully understood. Additionally, surfactants used in preparation of nano-co-crystals are likely to influence cocrystal solubility (24). Determination of the thermodynamic solubility is performable e.g. via the measurement of eutectic point concentrations. Additionally, methods of non *in situ* solubility analyses like centrifugation/filtration that have been widely applied in common formulations may not be suitable for nanocrystals, co-crystals and nano-co-crystals. A fraction of nanoparticles would exist in the supernatant after centrifugation and filtration; precipitation and dissolution occur simultaneously on the co-crystal surface. Thus, non *in situ* methods do not present sufficient kinetic solubility values in time and may result in incorrect values. To obtain a more realistic value for kinetic solubility and a deeper understanding of the formulation, *in situ* kinetic solubility analyses are superior to non *in situ* methods (25–27).

In our previous study, *in situ* kinetic solubility determination of different nanocrystals was successfully performed on the basis of UV-Vis spectroscopy (12). The equipment not only dynamically detects changes in solubility (as short as every 5 s), but the automated platform also provided a Tyndall-Rayleigh scattering correction to exclude scattering from undissolved particles and to obtain the absorbance of dissolved drug only. Furthermore, increased maximum kinetic solubilities were found with increased excess conditions, which was attributed to a higher amount of smaller nanocrystals whereas no plateau of this effect was revealed. The aim of this study was therefore to prepare nano-co-crystals and determine their kinetic solubility and dissolution rate *in situ*. Itraconazole (ITZ) and indomethacin (IND) were used as model drugs because of their low solubility and dissolution rate and ability to form nanocrystals or co-crystals (3, 16, 17). Efficient wet bead milling was applied to obtain nano-co-crystal formulations (28). Solid state and particle size of various co-crystals and nano-co-crystals were investigated, whereas crystallinity changes upon milling of raw drug powder and co-crystals were compared. The synergistic effect of nanocrystals and co-crystals on increased kinetic solubility was evaluated. In order to understand how nanocrystal technique promotes the kinetic solubility of co-crystals, further *in situ* kinetic solubility studies were conducted by comparing macro-, micro- and nano-co-crystals. Such detailed in situ investigation on the different generation of increased kinetic solubility of various sized co-crystals was not performed before and will clarify the creation of different maximum kinetic solubilities derived from different dissolution rates and their contribution to precipitation and the amount of dissolved drug. Furthermore, the maximum kinetic solubility of different nano-co-crystals was determined under different excess conditions.

## Materials and methods

### Materials

Itraconazole (BASF AG, Ludwigshafen, Germany), indomethacin (Fluka Chemie AG, Buchs, Switzerland), fumaric acid, succinic acid, nicotinamide, saccharin (Merck KGaA, Darmstadt, Germany), poloxamer 188, poloxamer 407, Tween 80, D-α-tocopheryl polyethylene glycol 1000 succinate (TPGS), polyvinylpyrrolidone K30 (PVP K30) (BASF SE, Ludwigshafen, Germany), sodium dodecyl sulfate (SDS) (Carl Roth GmbH & Co., Karlsruhe, Germany), hydroxypropylmethylcellulose E5 (HPMC E5) (Colorcon Ltd., Dartford Kent, UK), hydroxypropylcellulose (HPC) (Nisso Chemical Europe, Düsseldorf, Germany),, methanol, ethanol, dichloromethane, tetrahydrofuran, ethyl acetate (Carl Roth GmbH & Co., Karlsruhe, Germany), hydrochloric acid (HCl, Sigma Aldrich Chemie GmbH, Steinheim, Germany), ultrapurified water purified by a Milli-Q-apparatus (Millipore GmbH, Darmstadt, Germany), 0.25-0.35 mm zirkonium beads (SiLibeads^®^, Sigmund Lindner GmbH, Warmensteinach, Germany).

### Preparation of macro-co-crystals

Itraconazole (ITZ) co-crystal preparation was described previously (16). Briefly, 3.53 g ITZ in 20 mL chloroform and 0.29 g fumaric acid (FUM) or 0.30 g succinic acid (SUC) in 20 mL tetrahydrofuran were mixed in a bottle. The mixture was heated to 60 °C until the dissolution of all solids and then the bottles were sealed with a pierced parafilm to allow a slow evaporation. Once observing visible crystals, parafilm was removed to accelerate the evaporation. Finally, the solids were dried in a vacuum oven at 50 °C for 24 h until constant weight.

Indomethacin (IND) co-crystals were prepared by solvent evaporation (29). Briefly, 3.58 g of γ-form IND was mixed with 1.83 g saccharin (SAC) or 1.22 g of nicotinamide (NCT) in 100 mL ethyl acetate. The solutions were heated to 50 °C with continuous stirring until dissolution of all solids, then the solvent was evaporated in fume hood at room temperature followed by drying in a vacuum oven at 40 °C for 24 h until constant weight.

### Preparation of physical mixtures

Physical mixtures of ITZ and IND drug powder with their coformers were prepared by shaking the powder mixture together for 1 min in a glass vial or by mixing them gently with a pestle in a mortar.

### Preparation of nanocrystals, micro-co-crystals and nano-co-crystals by wet bead milling

Nanosuspensions of 5% (w/v) ITZ, IND and their macro-co-crystals were prepared by wet milling with yttria stabilized zirconium oxide beads with diameters of 0.3 mm using a ZentriMix 380 R (Andreas Hettich GmbH und Co. KG, Tuttlingen, Germany). The process and formulation screening was investigated by single factor experimental design and is described in detail in (30): in total 8 different stabilizers representing various stabilizer types were tested; PVP, HPMC, HPC, TPGS, Tween 80, SDS, poloxamer 188 and poloxamer 407; whereas PVP and HPMC were not able to form ITZ nanosuspensions and from the remaining stabilizers HPC, Tween 80 and poloxamer 407 were selected for further long time stability at different storage temperatures (4 °C, 25 °C and 40 °C). After 1 month of storage at 40 °C the following z-averages and PDIs were obtained: HPC (695 nm / 0.14), Tween 80 (685 nm / 0.15), poloxamer 407 (210 nm / 0.18) indicating poloxamer 407 as the stabilizer for further preparations including nano-co-crystal suspensions since its ability to stabilize also them and to ensure comparability during dissolution experiments between nanosuspensions and nano-co-crystal suspensions. The drug powders were added into 1% (w/v) poloxamer 407 solution and homogenized at 2000 rpm for 30 s with an Ultra Turrax T-25 (IKA^®^-Werke GmbH & Co. KG, Staufen, Germany); subsequently 5 mL suspension and milling beads in a ratio of 1:3 (w/w) were transferred into a 10 mL HDPE vial (iphas Pharma-Verpackung GmbH, Würselen, Germany) and milled at 750 rpm for 4 h. The cooling device was set to 0 °C which resulted in sample temperatures of approx. 15 °C after preparation. For comparison, ITZ micro-co-crystals were obtained with the same concentrations of drug and stabilizers as for the nanosuspensions; however, the milling time was shortened from 4 to 0.5 h. On the one hand this effective procedure was able to prepare particles in the lower μm-range enabling the investigation of size effects and on the other hand the preparation and formulation parameters were kept as similar as possible compared to the nanosuspensions ensuring comparability.

Directly after preparation, the suspensions were separated from beads by filtration through a sieve with a pore size of ∼20 μm, frozen at −80 °C and lyophilized (Alfa^®^ 2-4 LD Plus freeze-dryer, Martin Christ Gefriertrocknungsanlagen GmbH, Osterode am Harz, Germany). The lyophilization process was performed at −48 °C and 0.055 mbar.

### Characterization of nanocrystals, co-crystals and nano-co-crystals

#### Particle size analysis

The particle size of ITZ, IND drug powder, macro- and micro-co-crystals was measured by laser diffraction (Mastersizer^®^ 2000, Malvern Instruments Ltd., Malvern, UK, n = 5).

The particle size of the nanocrystals and nano-co-crystals after redispersion of the lyophilized powders in the drug saturated stabilizer solutions was measured by photon correlation spectroscopy (PCS) using a Zetasizer^®^ Nano ZS (Malvern Instruments Ltd., Malvern, UK, n = 3).

#### Light microscopy

Microscopic observations were performed using a polarized light microscope (Axioscope, Carl Zeiss Jena GmbH, Jena, Germany).

#### Differential scanning calorimetry (DSC)

Thermal analysis was conducted using a DSC (DSC 6000, PerkinElmer LAS GmbH, Rodgau, Germany) and data were analysed by Pyris software (PerkinElmer LAS GmbH, Rodgau, Germany). 5 ± 1 mg drug powder, physical mixture, macro-co-crystal, nanocrystal and nano-co-crystal freeze dried powder were accurately weighed in 50 μL aluminium pans with pierced lids. The samples were heated from 20 to 200 °C with a heating rate of 10 °C/min and then cooled to room temperature with a cooling rate of 20 °C/min. The test was performed under a continuously purged dry nitrogen atmosphere (flow rate 40 mL/min).

#### X-ray powder diffraction (XRPD)

The XRPD measurements were performed using Cu Kα radiation (λ = 0.154 nm) on a Bruker D8 Advance diffractometer (Bruker AXS GmbH, Karlsruhe, Germany) in the 2θ range from 10° to 80° with a step size of 0.01°.

### In situ *kinetic solubility studies*

The kinetic solubility experiments were performed by *in situ* measurements with Sirius^®^ inform (Sirius Analytical Instruments Ltd., Forest Row, UK). The cell path length of the probe tip was changed from 10 mm to 1 mm in order to prevent any particle scattering effect and to achieve suitable absorbance range (0.1-0.9). The absorbance was recorded by *in situ* UV-Vis spectroscopy from 200 to 700 nm with an interval of 60 s. During data analysis and evaluation, a broader time interval was considered to obtain a better visualization in some cases. Prior to the kinetic solubility test, the calculation of the amount of drug dissolved in the media was made by the software (Sirius^®^ inForm Refine) and took into account the molar extinction coefficient (MEC), which was determined through the UV-metric MEC/pKa-assay of Sirius^®^ inForm. A Tyndall-Rayleigh scattering correction was applied to the recorded spectra (Sirius^®^ inForm Refine) to exclude the scattering of undissolved particles and to obtain the absorbance of only dissolved drug (12). ITZ and IND drug powder, nanocrystals, macro-, micro- and nano-co-crystals powder were investigated under same excess conditions in dissolution media at 37 °C with 300 rpm paddle stirring rate to prevent floating of the powder. Specifically, with ITZ corresponding to 10 mg drug (40 times excess), all formulations were tested in 50 mL 0.1 N HCl (21), and in case of IND, corresponding to 8 mg drug (20 times excess), all formulations were investigated in 40 mL water (31). In order to determine the maximum kinetic solubility of nano-co-crystals, the kinetic solubility study was also performed under different excess (non-sink) conditions, with drug loadings 5-100 times higher than the drug equilibrium solubility.

### Stability studies of nano-co-crystal suspensions and powders

The stability studies of suspension and powder forms of nano-co-crystals were carried out at 4, 25 and 40 °C for 90 days wherein samples were analyzed at intervals of initial, 30 and 90 days. The particle size and PDI were measured as described in 2.5.1. The maximum kinetic solubility during dissolution were measured as described in Section 2.6.

## Results and discussion

### Preparation and characterization of drug nanocrystals, macro-, micro- and nano-co-crystals

ITZ forms co-crystals with a range of aliphatic dicarboxylic acids by solvent evaporation-induced crystallization (16, 32). Co-crystals were formed with fumaric acid and succinic acid as coformers (ITZ-FUM, ITZ-SUC). Subsequently, they were wet milled into the micro- and nano size range. Formulations were stabilized with poloxamer 407, a commonly used stabilizer in nanocrystal formulations (28, 33). ITZ powder and its two macro-co-crystals were in the 30-100 μm size range. After 0.5 h milling, the particle sizes of the two macro-co-crystals decreased to 10-20 μm. After 4 h, the particle sizes of ITZ-FUM and ITZ-SUC were around 400 nm with a narrow distribution (Table I).

**Table I:** Particle size characterization of itraconazole (ITZ) particles (±S.D., n = 3)

Sample	Size measurement, μm	Particle size after redispersion of freeze-dried powder	
	D50	z-average, nm	PDI
ITZ raw powder	50 ± 6	–	–
ITZ nanocrystal	–	223 ± 5	0.24 ± 0.02
ITZ-FUM macro	107 ± 5	–	–
ITZ-FUM micro	18 ± 2	–	–
ITZ-FUM nano	–	443 ± 7	0.35 ± 0.02
ITZ-SUC macro	36 ± 5	–	–
ITZ-SUC micro	13 ± 2	–	–
ITZ-SUC nano	–	455 ± 9	0.24 ± 0.01

**Fig. 1:**
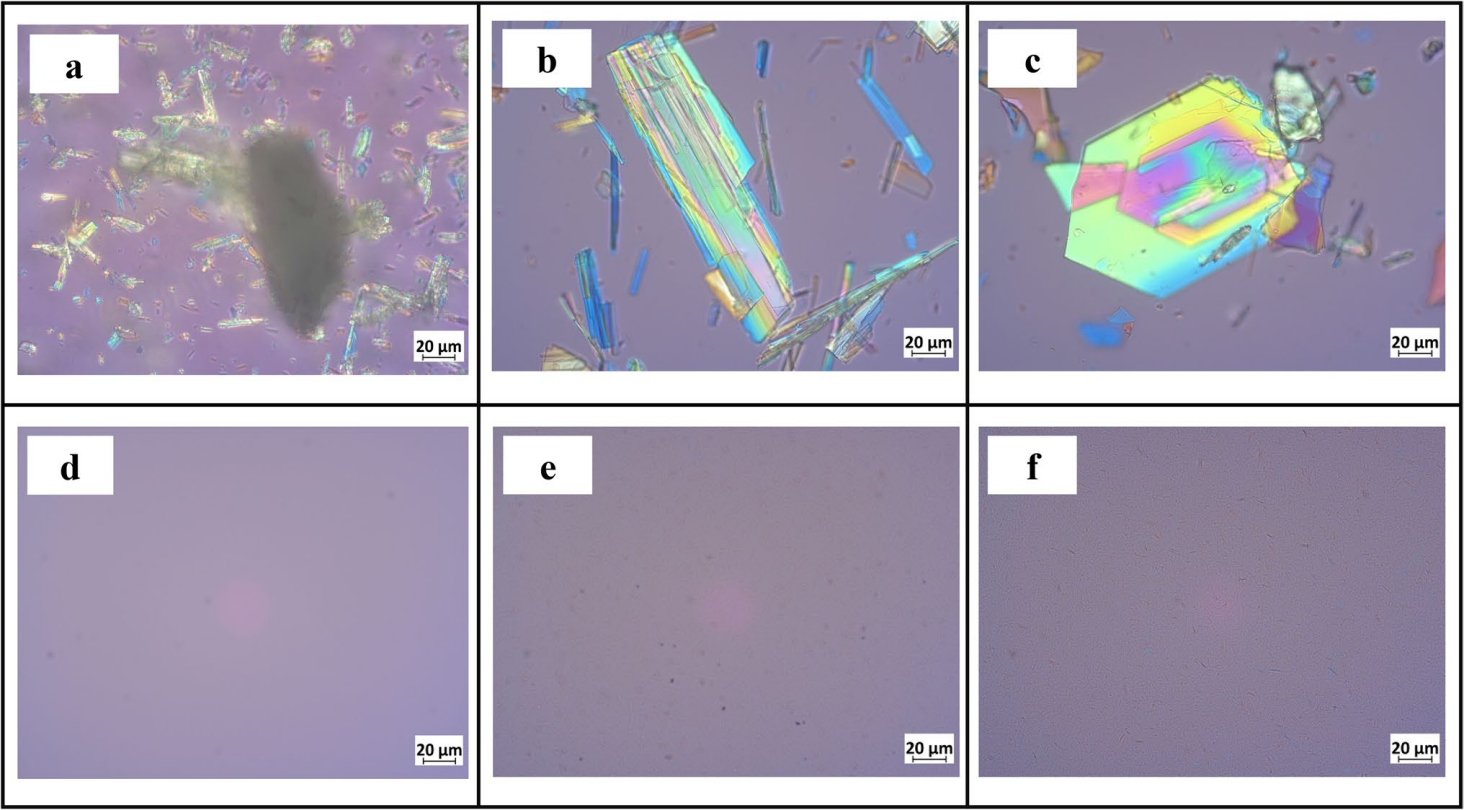
Optical microscopic pictures of (a) ITZ powder, (b) ITZ-FUM macro-co-crystal, (c) ITZ-SUC macro-co-crystal, (d) ITZ nanocrystal, (e) ITZ-FUM nano-co-crystal, (f) ITZ-SUC nano-co-crystal

The morphological evaluation displayed a substantial difference between ITZ powder and the two macro-co-crystals (Fig. 1a-c). The ITZ particles were irregular crystals and drug agglomerates formed by static electricity. In contrast, ITZ-FUM co-crystals were rod-shaped; and the ITZ-SUC co-crystals were hexagonal plate like, in accordance with the literature (34). No large particles were observed after milling, which was consistent with PCS measurements (Fig. 1d-f).

**Fig. 2:**
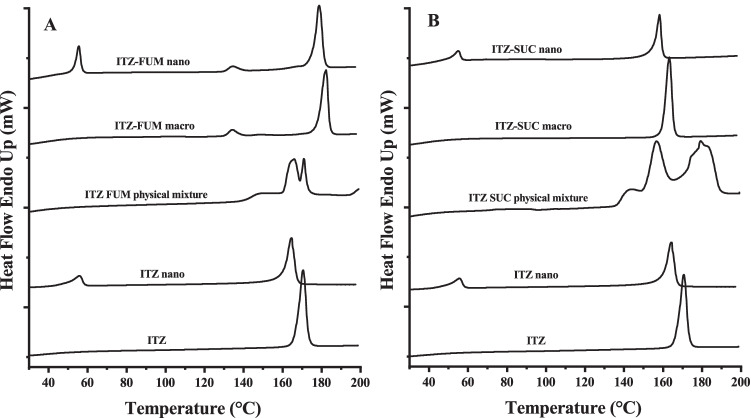
Differential scanning calorimetry profiles of different itraconazole (ITZ) formulations

The preservation of the solid state co-crystal structure, despite being subjected to high energy stress in aqueous media, must be confirmed after wet milling. ITZ-FUM macro-co-crystals exhibited an endothermic peak at 184 °C that was higher than the melting peak of ITZ (170 °C) and the physical mixture (165 °C) (Fig. 2A). ITZ-FUM macro-co-crystals showed a second small endothermic peak at around 135 °C, perhaps because a small part of ITZ and FUM did not form co-crystals or formed other isotropic crystal structures. ITZ-SUC macro-co-crystals displayed a single melting peak at 165 °C, which differed from the melting peak of ITZ and the endothermic melting peak of the physical mixture (157 °C and 180 °C) (Fig. 2B). After milling, a small peak at 55 °C indicated the small fraction of poloxamer 407 and the onset melting temperatures of ITZ-FUM and ITZ-SUC nano-co-crystals slightly shifted to lower temperatures compared to their macro-co-crystals (Fig. 2A, B). Either the stabilizer melted at lower temperatures and dissolved the drug to form eutectic mixtures, or a reduced degree of crystallinity might explain the melting peak shift (13, 35). ITZ-FUM macro-co-crystals exhibited the highest melting point potentially due to a stronger crystal lattice structure and resulted in less crystallinity reduction after milling into ITZ-FUM nano-co-crystals, resulting in the smallest peak shift compared to ITZ nanocrystals and ITZ-SUC nano-co-crystals.

**Fig. 3:**
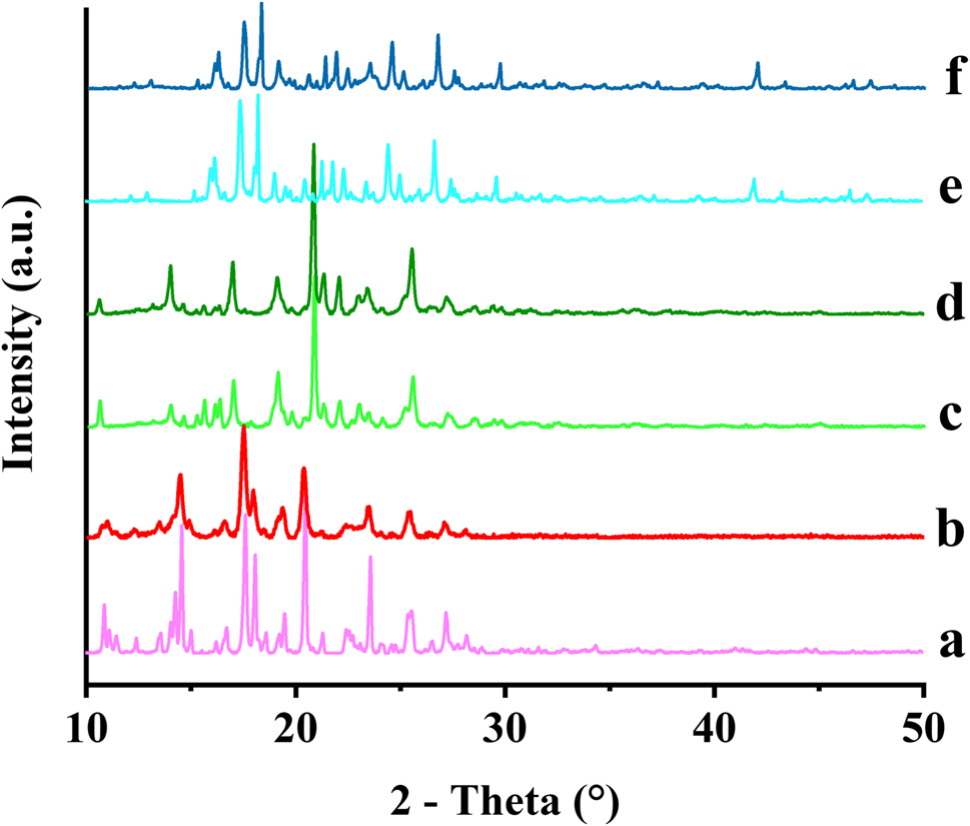
XRPD patterns of different ITZ formulations: (a) ITZ powder, (b) ITZ nanocrystal, (c) ITZ-FUM macro-co-crystal, (d) ITZ-FUM nano-co-crystal, (e) ITZ-SUC macro-co-crystal and (f) ITZ-SUC nano-co-crystal

The XRPD spectra of ITZ powder, ITZ-FUM and ITZ-SUC macro-co-crystals were evaluated to confirm the co-crystal structure and crystallinity before and after wet milling (Fig. 3). ITZ powder showed distinct crystalline peaks at 14.4, 17.4, 20.5, 23.5 and 25.7°, while ITZ-FUM and ITZ-SUC macro-co-crystals showed different crystalline peaks indicating different crystal structure (36). The XRPD patterns of ITZ nanocrystals and ITZ-FUM and ITZ-SUC nano-co-crystals did not change after wet milling, however, the peak intensity of all the nanocrystal formulations showed a slight decrease which indicated a slight reduction of crystallinity during wet milling. ITZ co-crystals were able to maintain the crystalline structure during wet milling process, one possible reason is their high binding energies in the crystal lattice, another reason is the presence of stabilizers that may alter the dissolution behaviour during wet milling. Combined with DSC analysis above, the co-crystal structure of both nano-co-crystals remained unchanged after wet milling.

Indomethacin (IND) powder and its two macro-co-crystals were all in the 30-70 μm size range. The mean particle sizes of the IND-SAC and IND-NCT nano-co-crystals were around 300 nm and with narrow particle size distributions (Table II).

**Table II:** Particle size characterization of indomethacin (IND) particles (±S.D., n = 3)

Sample	Size measurement, μm	Particle size after redispersion of freeze-dried powder	
	D50	z-average, nm	PDI
IND raw powder	70 ± 9	–	–
IND nanocrystal	–	163 ± 1	0.14 ± 0.02
IND-SAC macro	68 ± 10	–	–
IND-SAC nano	–	329 ± 10	0.20 ± 0.02
IND-NCT macro	29 ± 8	–	–
IND-NCT nano	–	280 ± 4	0.29 ± 0.01

**Fig. 4:**
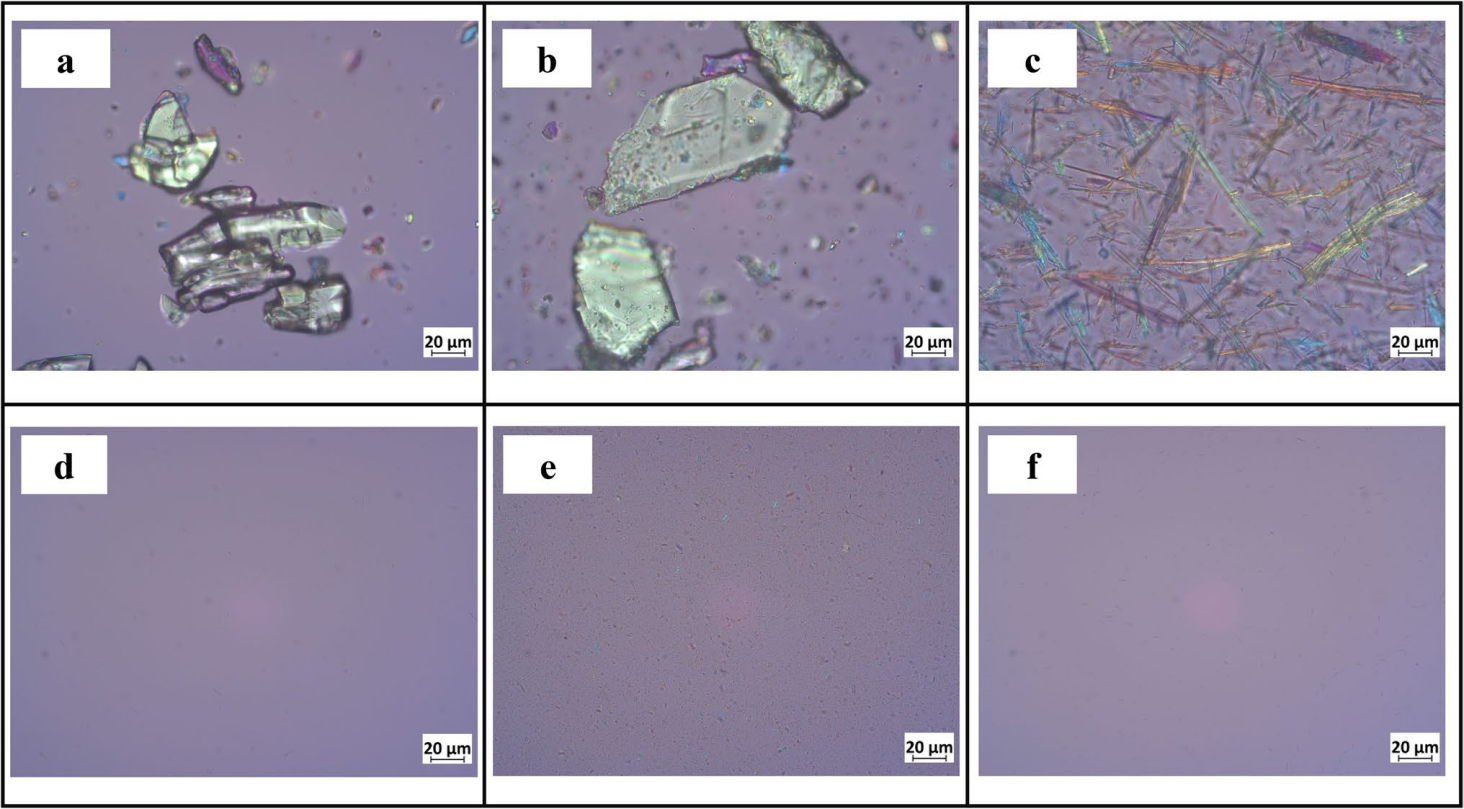
Optical microscopy pictures of (a) IND powder, (b) IND-SAC macro-co-crystal, (c) IND-NCT macro-co-crystal, (d) IND nanocrystal, (e) IND-SAC nano-co-crystal, (f) IND-NCT nano-co-crystal

The morphological evaluation displayed a substantial difference between IND powder and the two macro-co-crystals (Fig. 4). IND powder crystals were irregular. However, IND-SAC co-crystals were plate-like crystals and IND-NCT co-crystals were needle-like (Fig. 4a-c). After milling, large particles were not observed, which was consistent with particle size measurements (Fig. 4d-f).

**Fig. 5:**
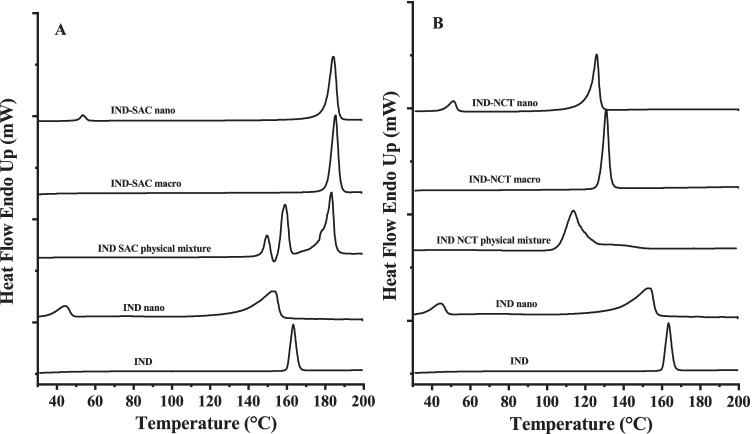
Differential scanning calorimetry profiles of different indomethacin (IND) formulations

The melting temperatures of IND powder, IND-SAC and IND-NCT macro-co-crystals were at 165 °C, 180 °C and 120 °C, respectively (Fig. 5). The physical mixture of IND with saccharin exhibited multi-melting transition points and the physical mixture of IND with nicotinamide exhibited overlaid melting peaks (Fig. 5). After wet milling, the small peak around 50 °C indicated a small fraction of poloxamer 407. Compared to IND powder, the endothermic melting peak of IND nanocrystals not only shifted to lower temperatures, but also turned broader (Fig. 5). IND was known to undergo polymorphism and partial amorphization during milling (31). The onset melting temperatures of IND-SAC and IND-NCT nano-co-crystals slightly shifted to lower temperatures without notable peak broadening (Fig. 5).

**Fig. 6:**
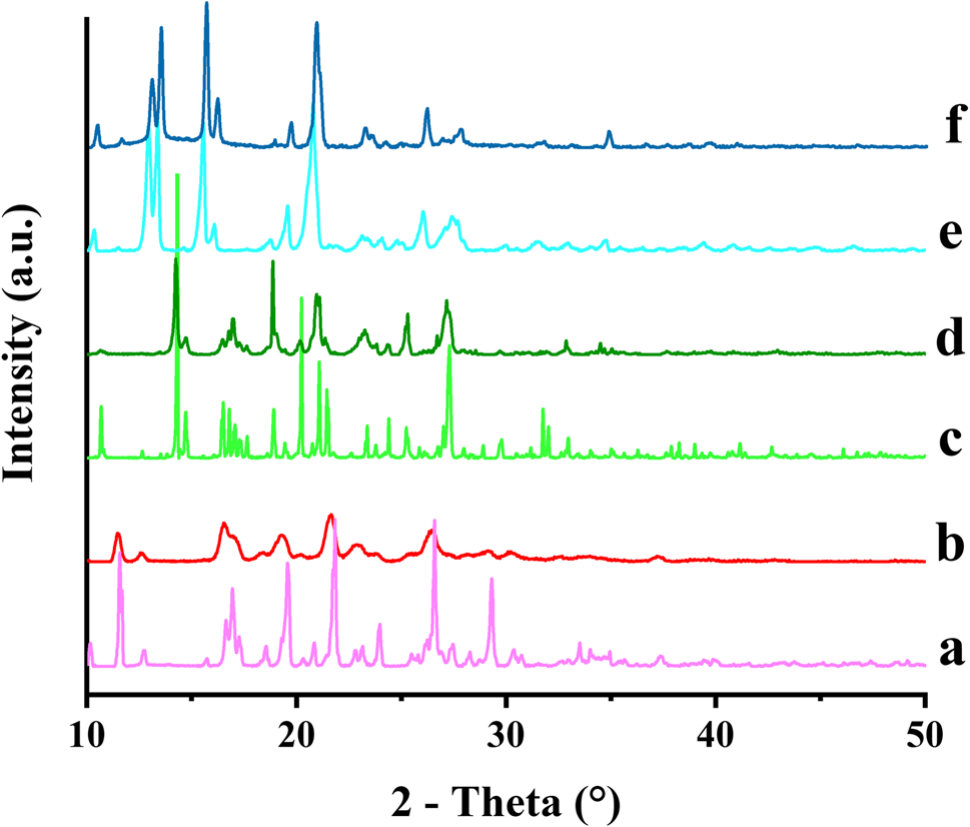
XRPD patterns of different indomethacin (IND) formulations: (a) IND powder, (b) IND nanocrystal, (c) IND-SAC macro-co-crystal, (d) IND-SAC nano-co-crystal, (e) IND-NCT macro-co-crystal and (f) IND-NCT nano-co-crystal

IND powder exhibited distinct peaks at 11.6, 19.6, 21.8, 26.6 and 29.3°, while IND-SAC macro-co-crystals exhibited characteristic peaks at 11.0, 14.3 and 24.9° (Fig. 6), consistent with literature (37, 38). In addition, IND-NCT macro-co-crystals demonstrated different characteristic peaks from IND raw powder and IND-SAC. After milling, the IND nanocrystal XRPD pattern indicated a considerable decrease in the intensity of crystalline peaks and the disappearance of some distinct peaks. Both nano-co-crystals presented similar crystalline characteristic peaks when compared to their macro-co-crystals, with only a slight decrease in intensity.

Compared to macro-co-crystals, the melting peak of both nano-co-crystals slightly shifted to lower temperature and the intensity of XRPD pattern decreased slightly. A reduction in crystallinity during wet milling was thus unavoidable. However, the transformation of API into its co-crystal form suggested a new strategy for the preparation of nano-formulations which were physically or chemically unstable during wet milling. Amorphization during nano milling was lower for IND co-crystals than for the drug powder. This was also observed during nano milling of ITZ powder and co-crystals, although the trend was not so pronounced. The formation of co-crystals may alter physical drug properties like hardness by crystal lattice structure transformation. The hydrogen bonding, which was the major interaction between the drug and coformers, may fix the structure of crystals and prevent the drugs’ hydrogen bonding sites from hydration with water during wet milling. Additionally, the altered dissolution behaviour of co-crystals which is for instance influenced by the presence of stabilizer might contribute to this observation. Co-crystal formulation was therefore a promising strategy to avoid phase transformation during wet milling processes.

### Comparison of dissolution profiles of drug powder, nanocrystals, macro- and nano-co-crystals

**Fig. 7:**
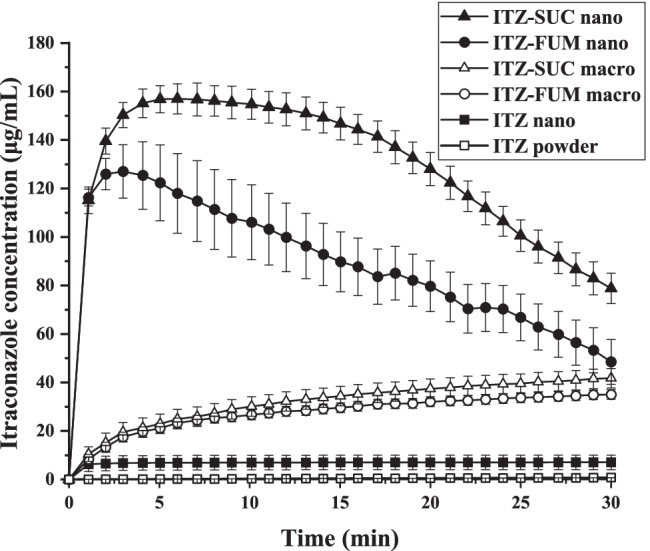
Dissolution profiles of itraconazole (ITZ) powder, ITZ nanocrystal, ITZ-FUM macro-co-crystal, ITZ-SUC macro-co-crystal, ITZ-FUM nano-co-crystal and ITZ-SUC nano-co-crystal

The kinetic solubility and dissolution rate of ITZ-FUM and ITZ-SUC nano-co-crystals were compared to ITZ powder, macro-co-crystals and nanocrystals in the presence of 0.004% poloxamer 407 (Fig. 7). Even though the influence of poloxamer 407 in this concentration to the solubility is neglectable it is important to keep this concentration constant to ensure comparability among various samples since it potentially improves the wettability which is followed by an increased dissolution rate (12). The concentration of crystalline ITZ was low up to 30 min (around 1 μg/mL) and reached a plateau of 5.1 μg/mL after 6 h. The solubility of nanocrystals was approx. 7.1 μg/mL, increased by a factor of 1.4 (12). No significant increase in crystalline drug nanosuspensions solubility was expected. Nanocrystals, however, still exhibited a superior dissolution rate as they reached the solubility plateau within 1 min. ITZ-FUM and ITZ-SUC macro-co-crystals resulted in a higher kinetic solubility of 35.1 μg/mL and 42.4 μg/mL after 30 min, with an increase by a factor of 6.9 and 8.3, respectively. ITZ-FUM and ITZ-SUC nano-co-crystals exhibited superior kinetic solubility and dissolution rate when compared to nanocrystals and macro-co-crystals. Specifically, ITZ-FUM and ITZ-SUC nano-co-crystals had a maximum kinetic solubility of 127.0 μg/mL and 156.9 μg/mL, respectively, with an increase by a factor of 24.9 and 30.8. Additionally, nano-co-crystals reached the plateau in less than 5 min, indicating that nanosized formulations owned superior dissolution rate due to their increased surface area. Nano-co-crystals successfully combined nanocrystal and co-crystal technologies to overcome the limitation of nanocrystals with regard to solubility improvement and of co-crystals with regard to rate of dissolution.

**Fig. 8:**
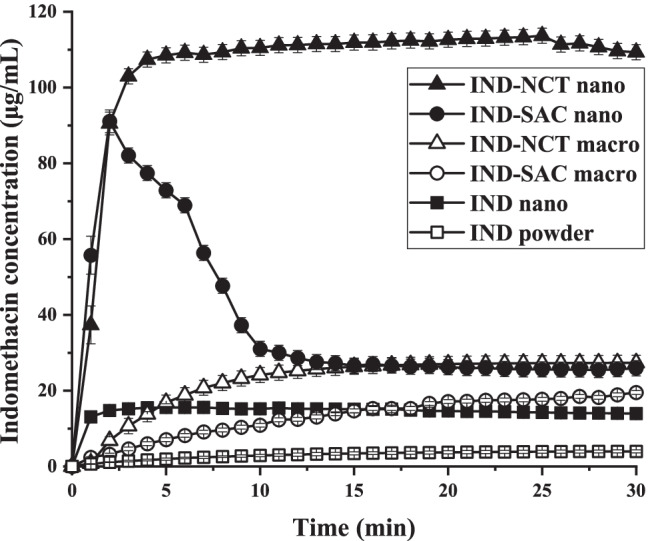
Dissolution profiles of indomethacin (IND) powder, IND nanocrystal, IND-SAC macro-co-crystal, IND-NCT macro-co-crystal, IND-SAC nano-co-crystal and IND-NCT nano-co-crystal

The kinetic solubility of IND powders, nanocrystals, macro- and nano-co-crystals in the presence of poloxamer was tested. The saturation solubility of IND powder and nanocrystal powder was 9.0 μg/mL after 5 h (equilibrium solubility) and 15.6 μg/mL after 1 min (Fig. 8). Compared to the drug powder, the solubility of IND nanocrystals increased by a factor of 1.7; IND-SAC and IND-NCT macro-co-crystals reached solubility plateaus of 21.3 μg/mL and 27.3 μg/mL, with an increase by a factor of 2.4 and 3.0. The reduction of the particle size of macro-co-crystals to the nano range strongly increased the kinetic solubility. The maximum kinetic solubility of IND-SAC and IND-NCT nano-co-crystals was 91.1 μg/mL and 112.2 μg/mL with an increase of 10.1 and 12.4. IND-SAC nano-co-crystals showed a short time at peak solubility and decreased solubility, which was considered as “spring and parachute”. In contrast, IND-NCT nano-co-crystals had a higher supersaturation level of 12.4 which was maintained for more than 30 min (39). IND may have greater interaction with NCT but less interaction with SAC after dissolution, thus explaining the longer “parachute time” with IND-NCT than with IND-SAC nano-co-crystals (39, 40). These *in situ* results indicated the synergistic effect of nanocrystals and co-crystals in the nano-co-crystal formulation, which each technology could not achieve independently.

### Effect of particle size on the dissolution profiles of ITZ co-crystals

**Fig. 9:**
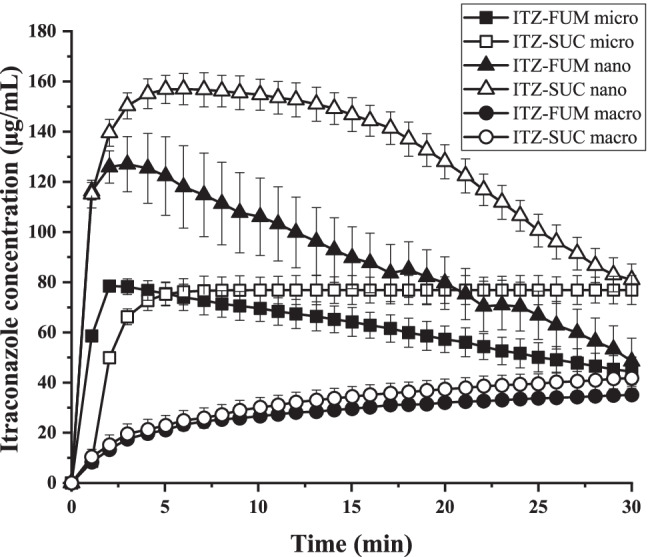
Dissolution profiles of itraconazole (ITZ) macro-, micro- and nano-co-crystal formulations

In order to further understand the mechanism of synergistic effect of nanocrystals and co-crystals with regard to increased kinetic solubility and dissolution rate, different co-crystal particle sizes were investigated with *in situ* kinetic solubility profiles. Same excess amounts (corresponding to 10 mg ITZ) of macro-, micro- and nano-co-crystals were added to the dissolution media and their kinetic solubility were analysed. As shown above, ITZ-FUM and ITZ-SUC macro-co-crystals resulted in a kinetic solubility of approximately 40 μg/mL. According to previous research, nanocrystal solubility was increased with minor factor (around 1.5) resulting from a small fraction of small nanoparticles and a reduction of crystallinity (11, 12). The theoretical kinetic solubility value of nano-co-crystals should not be more than 60 μg/mL (1.5 * 40 μg/mL). However, the experimental kinetic solubility of ITZ-FUM and ITZ-SUC nano-co-crystal (127 and 157 μg/mL) was more than twice higher than 60 μg/mL (Fig. 9). Upon water contact, the diffusion layer around co-crystal particles was highly concentrated and reaches a supersaturation state. The processes of dissolution and precipitation therefore occur simultaneously. The dissolution rate of macro-co-crystals was not high enough to overcome precipitation at the co-crystal surface, which prevented further dissolution (19, 20). By applying nanotechnology, the dissolution rate was highly promoted and outweighed the precipitation process, resulting in the highest maximum kinetic solubility. ITZ micro-co-crystals with medium particle size were compared to its macro- and nano-co-crystals. As expected, the kinetic solubility of micro-co-crystals was higher than of macro-co-crystals and lower than of nano-co-crystals. Therefore, the kinetic solubility of co-crystal formulations increased with reduced particle size (Fig. 9). The particle size can be further reduced by optimizing the preparation method and formulation. In general, nanocrystal technique effectively promoted the potential of co-crystal supersaturation effect by its superior dissolution rate as explained by the competition between dissolution rate and precipitation rate.

### Maximum kinetic solubility of nano-co-crystals

ITZ and IND nano-co-crystals were used to investigate the maximum kinetic solubility of nano-co-crystals under various excess conditions. As the influence of the small amount of poloxamer 407 on the solubility was negligible, the kinetic solubility profiles of nano-co-crystals were determined with drug loadings 5-100 times higher than the equilibrium solubility of the drug powder.

**Fig. 10:**
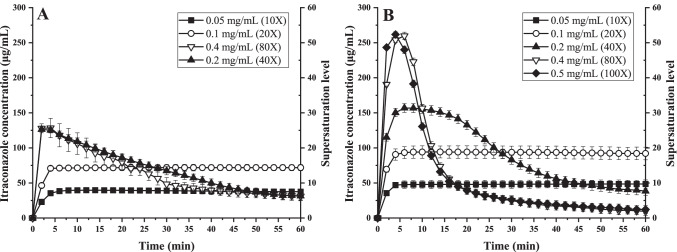
The maximum kinetic solubility achievable with increasing excess conditions of (A) ITZ-FUM nano-co-crystals and (B) ITZ-SUC nano-co-crystals

The maximum kinetic solubility of ITZ-FUM and ITZ-SUC nano-co-crystals was determined with 10, 20, 40, 80 and 10, 20, 40, 80, 100 times excess conditions. The solubility profiles were plotted using the supersaturation level as the right vertical axis (Fig. 10). The maximum supersaturation level of ITZ-FUM nano-co-crystals was approximately 26. The maximum kinetic solubility of ITZ-FUM nano-co-crystal was 127 μg/mL and did not further increase from 40 to 80 times excess (Fig. 10A). For ITZ-SUC nano-co-crystals, the maximum supersaturation level was approximately 55. The maximum kinetic solubility of ITZ-SUC nano-co-crystal was 260 μg/mL and did not further increase from 80 to 100 times excess (Fig. 10B). ITZ-SUC nano-co-crystals had much higher kinetic solubility than ITZ-FUM nano-co-crystals under the same excess conditions. Since succinic acid has a remarkably higher solubility than fumaric acid in the dissolution media, succinic acid separates faster from the co-crystal structure and causes higher crystal arrangement disruption, hence achieving higher supersaturated solubility.

**Fig. 11:**
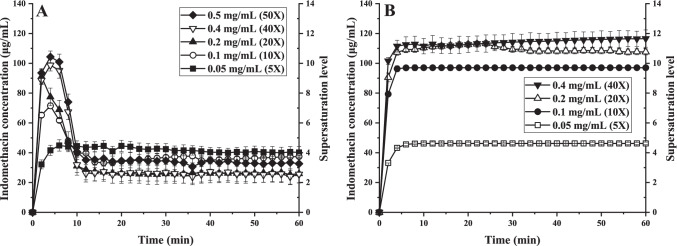
The maximum kinetic solubility achievable with increasing excess conditions of (A) IND-SAC nano-co-crystals and (B) IND-NCT nano-co-crystals

The maximum kinetic solubility of IND-SAC and IND-NCT nano-co-crystals was determined with 5, 10, 20, 40, 50 and 5, 10, 20, 40 times excess conditions. The maximum supersaturation level of IND-SAC and IND-NCT nano-co-crystals was approximately 12 and 13 (Fig. 11A, B). The addition of more nano-co-crystals to higher excess did not increase the kinetic solubility further in both cases. Those revealed plateaus of the maximum kinetic solubility regarding increased excess conditions were remarkable since previous studies did not demonstrate them (12). Even though the amount of small nano-co-crystals was higher with increased excess conditions this resulted not in a higher kinetic solubility, demonstrating that the beneficial effect of an increased dissolution rate due to nanoization is limited.

For all nano-co-crystals, lower excess conditions exhibited longer supersaturation maintaining time. At 10- and 20-times excess condition, ITZ-FUM and ITZ-SUC nano-co-crystals maintained supersaturation for more than 1 h, while at 40 times excess, precipitation started after 5 min. IND-SAC nano-co-crystals maintained supersaturation for more than 1 h at 5 times excess, while all the higher excess started precipitating in less than 5 min. The exception was IND-NCT nano-co-crystals, where no precipitation was observed under all excess conditions. IND-NCT could form a stable complex during dissolution and precipitation was expected for longer observation time. Nano-co-crystal precipitation induction time decreased as excess conditions increased. At the step of nucleation and crystal growth in a supersaturated system, each supersaturated solution had a maximum supersaturation limit, defined as metastable zone width. When the concentration exceeded this limitation, precipitation occurred; whereas if the concentration was within the metastable zone, the induction time for precipitation was longer (41). Additionally, rapid and heterogeneous precipitation occurred as more undissolved particles in higher excess conditions acted as crystallization nucleus in the supersaturated system (42). Comparing ITZ and IND nano-co-crystals, under 40 times excess, IND nano-co-crystals only achieved the supersaturation level of around 10, while ITZ-FUM and ITZ-SUC nano-co-crystals achieved the supersaturation level of 26 and 30. Different compounds had different supersaturation properties and ITZ exhibited higher supersaturation properties than IND (43). Precipitation and recrystallization clearly existed in nano-co-crystal formulations. Further investigations of these precipitated residues would be worth to reveal their solid state form. Potentially those precipitates represent a confirmation with a low solubility and with a large particle size combined with a low dissolution rate refusing the intended improved bioavailability. Thus, further studies are required to extend the time of increased kinetic solubility. One potential approach is *in situ* recrystallization inhibitors investigation (44).

### Stability studies of nano-co-crystal suspension and powder

The stability data of suspension and powder form of ITZ-FUM, ITZ-SUC, IND-SAC and IND-NCT nano-co-crystals over a period of 90 days at different temperature were summarized respectively (data shown in Table S1-S4 of Supplementary information). ITZ-FUM nano-co-crystal suspensions lost its nano size after 30 days at 4 °C due to both crystal growth and particles aggregation, particles formed a cake sediment and were no longer disperse. The particle size and PDI of ITZ-SUC, IND-SAC and IND-NCT nano-co-crystal suspensions slightly increased with increase of storage temperature after 30 days; while after 90 days, non-dispersible aggregates was observed indicated the particle aggregation. The maximum kinetic solubility of suspension form decreased with storage time and increased storage temperature. The powder form of all nano-co-crystals was relatively stable with regard to not only particle size and PDI but also maximum kinetic solubility during at least 90 days storage at 4, 25 and 40 °C. Nanosuspension were more prone to physical instability phenomena like Ostwald ripening or agglomeration during storage compared to its powder forms. Moreover, the suspension form was more susceptible to microbial instability. Thus, nano-co-crystal suspensions may have to be converted into solid dosage forms for stability reasons.

## Conclusions

Four itraconazole and indomethacin nano-co-crystals with mean particle diameters of around 450 nm were successfully prepared for the first time. Solid-state characterization suggested that the transformation of drug powder into its co-crystal form could be a new strategy for the preparation of nano-formulations which normally were physically or chemically unstable during wet milling. *In situ* kinetic solubility studies indicated that nano-co-crystals showed remarkably increased kinetic solubility and dissolution rates compared to nanocrystals and co-crystals. The combination of co-crystals and nanocrystals could potentially overcome the limitation of nanocrystals with regard to the neglectable absolute solubility improvement, whereas relative increases depend on the inherent solubility of the drug, and the limitation of co-crystal with regard to dissolution rate improvement. Nanosizing efficiently promoted the potential of co-crystal solubilization by its superior dissolution rate. Therefore, this combination approach which adds nanosizing to co-crystallization was selected and considered suitable to potentially result in an increased bioavailability, which would be not possible by each single technique only. The maximum kinetic solubility of nano-co-crystals was determined under different excess conditions and reached a plateau. The optimization of the preparation process and *in situ* investigation of recrystallization inhibitors which extended the kinetic solubility time of nano-co-crystals should be analysed in future studies.

### Acknowledgements and Disclosures

We would like to thank Dr. Kang Dong (Department of Structure and Dynamics of Energy Materials, Technische Universität Berlin) for the assistance in XRPD analysis.

## Supplementary Information

ESM 1 (DOCX 24 kb)
