# Supplementary material for: Combination of co-crystal and nanocrystal techniques to improve the solubility and dissolution rate of poorly soluble drugs
Source: Pharm Res. 2022 May 12;39(5):949–61. doi: 10.1007/s11095-022-03243-9 (PMC9160134; doi:10.1007/s11095-022-03243-9)
Supplement: Supplementary file 1 — (DOCX 24 kb) [file 11095_2022_3243_MOESM1_ESM.docx]

**Supplementary Materials**

**Combination of co-crystal and nanocrystal techniques to improve the solubility and dissolution rate of poorly soluble drugs**

Zun Huang, Sven Staufenbiel, Roland Bodmeier*

College of Pharmacy, Freie Universität Berlin, Kelchstr. 31, 12169 Berlin, Germany

**Table S1.** Stability data of ITZ-FUM nano-co-crystal suspension and powder (±S.D., n = 3).

|  |  | Initial | 4 °C | | 25 °C | | 40 °C | |
| --- | --- | --- | --- | --- | --- | --- | --- | --- |
|  |  |  | 30 d | 90 d | 30 d | 90 d | 30 d | 90 d |
| Suspension | z-average, nm | 343 ± 7 | - | - | - | - | - | - |
|  | PDI | 0.36 ± 0.03 | - | - | - | - | - | - |
|  | C_max_, µg/mL | 112 ± 2 | 79 ± 3 | 70 ± 5 | 66 ± 1 | 58 ± 3 | 39 ± 2 | 30 ± 1 |
| Powder | z-average, nm | 443 ± 7 | 406 ± 10 | 456 ± 5 | 464 ± 8 | 440 ± 12 | 435 ± 3 | 428 ± 6 |
|  | PDI | 0.35 ± 0.02 | 0.32 ± 0.03 | 0.31 ± 0.01 | 0.42 ± 0.01 | 0.40 ± 0.02 | 0.38 ± 0.01 | 0.37 ± 0.02 |
|  | C_max_, µg/mL | 108 ± 5 | 107 ± 6 | 107 ± 5 | 108 ± 7 | 107 ± 3 | 106 ± 4 | 92 ± 10 |

**Table S2.** Stability data of ITZ-SUC nano-co-crystal suspension and powder (±S.D., n = 3).

|  |  | Initial | 4 °C | | 25 °C | | 40 °C | |
| --- | --- | --- | --- | --- | --- | --- | --- | --- |
|  |  |  | 30 d | 90 d | 30 d | 90 d | 30 d | 90 d |
| Suspension | z-average, nm | 294 ± 6 | 283 ± 5 | - | 470 ± 10 | - | 913 ± 52 | - |
|  | PDI | 0.29 ± 0.03 | 0.30 ± 0.02 | - | 0.20 ± 0.02 | - | 0.46 ± 0.05 | - |
|  | C_max_, µg/mL | 150 ± 10 | 154 ± 8 | 54 ± 9 | 66 ± 1 | 58 ± 3 | 39 ± 2 | 30 ± 1 |
| Powder | z-average, nm | 355 ± 9 | 412 ± 6 | 435 ± 5 | 446 ± 10 | 433 ± 6 | 418 ± 12 | 509 ± 22 |
|  | PDI | 0.24 ± 0.01 | 0.25 ± 0.01 | 0.28 ± 0.02 | 0.30 ± 0.01 | 0.30 ± 0.02 | 0.33 ± 0.01 | 0.39 ± 0.05 |
|  | C_max_, µg/mL | 157 ± 5 | 154 ± 3 | 151 ± 3 | 154 ± 10 | 158 ± 9 | 158 ± 4 | 145 ± 10 |

**Table S3.** Stability data of IND-SAC nano-co-crystal suspension and powder (±S.D., n = 3).

|  |  | Initial | 4 °C | | 25 °C | | 40 °C | |
| --- | --- | --- | --- | --- | --- | --- | --- | --- |
|  |  |  | 30 d | 90 d | 30 d | 90 d | 30 d | 90 d |
| Suspension | z-average, nm | 322 ± 6 | 459 ± 23 | - | 418 ± 15 | - | 328 ± 12 | - |
|  | PDI | 0.15 ± 0.01 | 0.43 ± 0.05 | - | 0.38 ± 0.04 | - | 0.29 ± 0.05 | - |
|  | C_max_, µg/mL | 120 ± 8 | 100 ± 8 | 35 ± 5 | 86 ± 6 | 25 ± 2 | 62 ± 2 | 30 ± 3 |
| Powder | z-average, nm | 329 ± 10 | 322 ± 9 | 435 ± 5 | 364 ± 10 | 503 ± 6 | 411 ± 9 | 483 ± 22 |
|  | PDI | 0.20 ± 0.02 | 0.21 ± 0.01 | 0.28 ± 0.02 | 0.30 ± 0.03 | 0.35 ± 0.03 | 0.33 ± 0.02 | 0.36 ± 0.04 |
|  | C_max_, µg/mL | 110 ± 10 | 114 ± 5 | 101 ± 4 | 105 ± 5 | 100 ± 11 | 108 ± 6 | 98 ± 2 |

**Table S4.** Stability data of IND-NCT nano-co-crystal suspension and powder (±S.D., n = 3).

|  |  | Initial | 4 °C | | 25 °C | | 40 °C | |
| --- | --- | --- | --- | --- | --- | --- | --- | --- |
|  |  |  | 30 d | 90 d | 30 d | 90 d | 30 d | 90 d |
| Suspension | z-average, nm | 294 ± 6 | 396 ± 8 | - | 500 ± 7 | - | 782 ± 12 | - |
|  | PDI | 0.29 ± 0.03 | 0.24 ± 0.01 | - | 0.26 ± 0.02 | - | 0.22 ± 0.01 | - |
|  | C_max_, µg/mL | 125 ± 2 | 110 ± 4 | 38 ± 5 | 86 ± 5 | 30 ± 1 | 78 ± 10 | 20 ± 2 |
| Powder | z-average, nm | 280 ± 4 | 321 ± 5 | 433 ± 9 | 303 ± 4 | 436 ± 10 | 395 ± 8 | 409 ± 9 |
|  | PDI | 0.29 ± 0.01 | 0.26 ± 0.02 | 0.28 ± 0.02 | 0.25 ± 0.01 | 0.30 ± 0.02 | 0.30 ± 0.03 | 0.29 ± 0.02 |
|  | C_max_, µg/mL | 106 ± 4 | 103 ± 2 | 97 ± 3 | 104 ± 10 | 98 ± 9 | 100 ± 5 | 95 ± 6 |
